# Effects of Cytochrome P450 and Transporter Polymorphisms on the Bioavailability and Safety of Dutasteride and Tamsulosin

**DOI:** 10.3389/fphar.2021.718281

**Published:** 2021-10-07

**Authors:** Gonzalo Villapalos-García, Pablo Zubiaur, Marcos Navares-Gómez, Miriam Saiz-Rodríguez, Gina Mejía-Abril, Samuel Martín-Vílchez, Manuel Román, Dolores Ochoa, Francisco Abad-Santos

**Affiliations:** ^1^ Clinical Pharmacology Department, School of Medicine, Hospital Universitario de La Princesa, Instituto Teófilo Hernando, Instituto de Investigación Sanitaria La Princesa (IP), Universidad Autónoma de Madrid, Madrid, Spain; ^2^ Research Unit of Hospital Universitario de Burgos (HUBU), Burgos, Spain; ^3^ Unidad de Investigación Clínica y Ensayos Clínicos (UICEC) Hospital Universitario de La Princesa, Platform SCReN (Spanish Clinical Research Network), Instituto de Investigación Sanitaria La Princesa (IP), Madrid, Spain; ^4^ Centro de Investigación Biomédica en Red de Enfermedades Hepáticas y Digestivas (CIBERehd), Instituto de Salud Carlos III, Madrid, Spain

**Keywords:** dutasteride, tamsulosin, pharmacogenetics, pharmacokinetics, *CYP2D6*, *CYP3A4*

## Abstract

Dutasteride and tamsulosin are one of the first-line combination therapies for the management of benign prostatic hyperplasia (BPH). Despite being more effective than monotherapies, they produce frequent adverse drug reactions (ADRs). Institutions such as Food and Drug Administration and European Medicines Agency recommend precaution with *CYP2D6* poor metabolizers (PMs) that receive *CYP3A4* inhibitors and tamsulosin. However, no specific pharmacogenetic guideline exists for tamsulosin. Furthermore, to date, no pharmacogenetic information is available for dutasteride. Henceforth, we studied the pharmacokinetics and safety of dutasteride/tamsulosin 0.5 mg/0.4 mg capsules according to 76 polymorphisms in 17 candidate pharmacogenes. The study population comprised 79 healthy male volunteers enrolled in three bioequivalence, phase-I, crossover, open, randomized clinical trials with different study designs: the first was single dose in fed state, the second was a single dose in fasting state, and the third was a multiple dose. As key findings, *CYP2D6* PMs (i.e., *4/*4 and *4/*5 subjects) and intermediate metabolizers (IMs) (i.e., *1/*4, *1/*5, *4/*15 individuals) presented higher AUC (*p* = 0.004), higher t_1/2_ (*p* = 0.008), and lower Cl/F (*p* = 0.006) when compared with NMs (*1/*1 individuals) and UMs (1/*1 × 2 individuals) after multiple testing correction. Moreover, fed volunteers showed significantly higher t_max_ than fasting individuals. Nominally significant associations were observed between dutasteride exposure and *CYP3A4* and *CYP3A5* genotype and between tamsulosin and *ABCG2*, *CYP3A5*, and *SLC22A1* genotypes. No association between the occurrence of adverse drug reactions and genotype was observed. Nonetheless, higher incidence of adverse events was found in a multiple-dose clinical trial. Based on our results, we suggest that dose adjustments for PMs and UMs could be considered to ensure drug safety and effectiveness, respectively. Further studies are warranted to confirm other pharmacogenetic associations.

## Introduction

Dutasteride and tamsulosin are one of the first-line combination therapies for the management of benign prostatic hyperplasia (BPH). Combination therapy is frequent in BPH patients, due to difficulties in reaching effectiveness with single treatments (Lerner et al., 2021a; Lerner et al., 2021b).

Dutasteride belongs to 5-*α* reductase inhibitors (5-ARIs), which prevent dihydrotestosterone production and, consequently, delay prostatic tissue growth. It is administered by oral route. It presents 60% oral bioavailability, and its median time to reach maximum plasma concentration (t_max_) is around 3 h (1–10 h range) after the administration of 0.5 mg single dose. Dutasteride shows a volume of distribution (Vd) of 300–500 L and a high plasma protein binding (>99.5%). Its elimination is dose-dependent. At single doses lower than 5 mg, dutasteride clearance is rapidly performed, with a shorter half-life (t_1/2_) of 3–9 days. However, at 0.5 mg daily doses, the elimination is slower, reaching a t_1/2_ of 3–5 weeks. It is extensively metabolized by cytochrome P450 (*CYP*) isoforms *CYP3A4* and *CYP3A5* into four major metabolites: two of them less active than dutasteride and two other that are similarly active to the parent drug. They are primarily excreted in stools and marginally in urine; only between 1 and 15.4% of the dutasteride dose is excreted unmetabolized in feces. It has been also reported that dutasteride is not metabolized *in vitro* by human cytochrome P450 isoenzymes *CYP1A2, CYP2A6, CYP2B6, CYP2C8, CYP2C9, CYP2C19, CYP2D6*, and *CYP2E1* (FDA, 2010).

Tamsulosin belongs to α-1 receptor antagonists (ARAs). This family of drugs reduces the sympathetic tone of smooth muscle in the prostate and bladder neck, facilitating urine expulsion. Tamsulosin is likewise administered orally and presents >90% oral bioavailability, linear pharmacokinetics, Vd of 16 L, and exhibits high plasmatic protein binding (94–99%). After the administration of 0.4 mg single-dose, the median t_max_ is 6 h (2–24 h range). It has a median t_1/2_ of 10–13 h both in single-dose and multiple-dose regimens. It is 90% metabolized by mainly *CYP3A4* and *CYP2D6*. The remaining unaltered tamsulosin (i.e., approximately 10% of the administered dose) is excreted in urine [Agencia Española del Medicamento y Productos Sanitarios (AEMPS), 2015].

Despite being more effective than monotherapy, combination therapies usually cause greater adverse drug reactions (ADRs) (Greco and McVary, 2008). Dutasteride/tamsulosin treatment may produce dizziness, erectile dysfunction, decreased libido, retrograde ejaculation, and breast alterations [Agencia Española del Medicamento y Productos Sanitarios (AEMPS), 2015]. In addition to the adverse events, underdosing can determine a lack of treatment effectiveness. It is, therefore, important to improve the effectiveness and tolerability of current therapies by means of individualized approaches. Genetic variants in genes encoding for drug metabolizing enzymes, transporters, or drug targets affect drug pharmacokinetics and pharmacodynamics, which relates to ADR occurrence and drug exposure. Notably, tamsulosin Food and Drug Administration (FDA) drug label includes an assortment according to patient pharmacogenetics. Particularly, caution should be exercised for *CYP2D6* poor metabolizers (PMs) treated with *CYP3A4* moderate inhibitors, for the risk of elevated drug blood levels (FDA, 2010). No other high level of evidence pharmacogenetic information is available for tamsulosin. Additionally, no pharmacogenetics information related to dutasteride is known.

Hence, our goal was to conduct a candidate gene pharmacogenetic study evaluating 76 polymorphisms in 17 pharmacogenes, including *CYP1A2, CYP2A6, CYP2B6, CYP2C19, CYP2C8, CYP2C9, CYP2D6, CYP3A4, CYP3A5,* and *CYP4F2* and transporters such as *ABCB1, ABCC2, ABCG2, SLC22A1, SLC28A3, SLCO1B1,* and *UGT1A1* in healthy volunteers participating in bioequivalence clinical trials.

## Materials and Methods

### Study Population

The study population was enrolled in three bioequivalence clinical trials testing two different formulations of dutasteride/tamsulosin 0.5 mg/0.4 mg hard capsules. Each clinical trial comprised 36 individuals. The number of volunteers who completed the three clinical trials and that provided their informed consent for the pharmacogenetic study was 88 out of 108. Nine of them were duplicates, i.e., they participated in two out of the three clinical trials, and were thence excluded from the repeated pharmacogenetic studies. Thus, the total number of volunteers that participated in this work was 79.

The clinical trials were performed at the Clinical Trial Unit of Hospital Universitario de La Princesa (UECHUP) (Madrid, Spain). Inclusion and exclusion criteria were common to the three clinical trials. They involved healthy males aged from 18 to 55 years old, who were either surgically sterile or that agreed to use double efficient contraceptive methods and that committed to avoid sperm donation for at least 6 months after the first administration of the drug. Exclusion criteria comprised any organic or psychic condition, previous use of prescription pharmacological treatment, body mass index (BMI) outside of the 18–30 kg/m^2^ range, consumption of abuse drugs, alcohol, or tobacco, blood donation in the previous month before starting the trial, and history of swallowing problems.

### Study Design

The reference formulation used in the clinical trials was Duodart® (tamsulosin/dutasteride 0.5/0.4 mg, GlaxoSmithKline, England), which was also used for the pharmacogenetic study. The three clinical trials presented different study designs. They were bioequivalence, phase-I, crossover, open, randomized clinical trials. They were blinded for the analytical determination of dutasteride and tamsulosin plasma levels. They differed in the dose regimen and the feeding conditions. In the first one, a single dose was administered under fed conditions (S1) (Supplementary Figure S1); in the second, a single dose was administered under fasting conditions (S2) (Supplementary Figure S2); in the last one, eight doses were administered during eight consecutive days under fed conditions (M) (Supplementary Figure S3).

S1 (fed-state) and S2 (fasting-state) studies consisted of a single oral dose of Duodart® or a test formulation administered in two periods to 36 subjects, respectively (*n* = 72). Both formulations contained dutasteride 0.5 mg/tamsulosin 0.4 mg. Volunteers were hospitalized from 10 h before to 24 h after dosing. Administration of the drug was done by investigators in the Clinical Trials Unit of the Hospital Universitario de la Princesa (UECHUP), and individuals were checked each time they swallowed the capsule. Three of them were excluded as did not complete the second period (*n* = 69). A 28-day washout period was scheduled between periods. Drug administration was established 10 min after a high-fat breakfast in S1 and 10 h after their last meal and 5 h before their next in S2. After drug intake, 23 blood samples were collected from each volunteer at 0 h (before receiving the drug), 0.5, 1, 1.5, 2, 2.5, 3, 3.5, 4, 5, 6, 7, 8, 9, 10, 12, 14, 16, 20, 24, 32, 48, and 72 h after the administration of the drug. Tamsulosin and dutasteride plasma concentrations were quantified. Likewise, M (multiple-dose) comprised 36 subjects who received Duodart® or a test formulation during eight days. 10 h before the last drug administration (i.e., the eighth dose), they were hospitalized until 24 h after dosing. Only tamsulosin plasma concentrations were quantified in this multiple-dose study. Two volunteers were excluded from the bioequivalence analysis as they did not complete the second period (*n* = 34). Periods were separated by a 7-day washout period. Every day, the volunteers visited the UECHUP to provide a trough blood sample (i.e., a total of seven blood samples) and to receive a standard breakfast and the dose. Drug intake was established 10 min after having breakfast. Afterward, on day 8, they were hospitalized. They received the drug 30 min before dosing and after fasting for 10 h. Then, 23 blood samples were obtained from each volunteer at 0 h (before receiving the drug), 0.5, 1, 1.5, 2, 2.5, 3, 3.5, 4, 5, 6, 7, 8, 9, 10, 12, 14, 16, 20, 24, 32, 48, and 72 h after the administration of the drug.

Clinical laboratory analyses and dutasteride and tamsulosin plasma level determinations were outsourced in the three clinical trials. During periods, samples were frozen at −20°C until their shipment to an external laboratory. Drug determinations were performed after liquid-liquid extraction by high-performance liquid chromatography coupled with mass spectrometry (LC-MS) with a lower limit of quantification (LLOQ) of 50.00 pg/ml for dutasteride and 99.80 pg/ml for tamsulosin.

The race or biogeographic origin variable was self-reported by healthy volunteers as well as their biological sex and age. Weight and height were measured during the screening to assess inclusion criteria.

### Pharmacokinetic Analyses

Pharmacokinetic parameters were calculated using CERTARA Phoenix WinNonlin Professional software version 7.0 (Certara United States, Princeton, NJ, United States) with a noncompartmental method for both drugs in S1 and S2 trials and for tamsulosin in M. In S1 and S2, the area under the curve (AUC) between 0 and 72 h (AUC_t_) was calculated with the linear trapezoidal rule. The AUC between 72 h and infinite (AUC_∞_) was estimated as C_t_/k_e_ (C_t_ being the drug plasma concentration at 72 h and k_e_ being the terminal rate constant, calculated by linear regression of the log-linear part of the concentration–time curve). The AUC between 0 and ∞ was calculated as AUC_t_ + AUC_t-∞_ (AUC_∞_). In M, the AUC at steady state, i.e., between the eighth drug administration and 24 h later (AUC_τ_), was similarly calculated with the linear trapezoidal rule. In the three clinical trials, the maximum plasma concentration (C_max_) and time to reach C_max_ (t_max_) were observed directly; the half-life (t_1/2_) was calculated as ln2/k_e_; clearance (Cl) was calculated as dose divided by AUC_∞_ or AUC_τ_; and volume of distribution (Vd) was estimated as Cl/k_e_. The minimum concentration in the steady state (C_min_) was directly observed in the multiple-dose clinical trial.

### Genotyping

DNA extraction from peripheral venous blood was performed in a MagNa Pure System (Roche Applied Science, United States). DNA concentration was measured with a Qubit 3.0 Fluorometer (ThermoFisher, United States). The genotyping was performed with a custom TaqMan® OpenArray® panel (Thermo Fisher Scientific, United States) in a QuantStudio 12k Flex real-time PCR system (Thermo Fisher Scientific, United States). Volunteers were genotyped for variants in genes potentially related to dutasteride/tamsulosin absorption, distribution, metabolism, and excretion, based on most important pharmacogenes and the literature (FDA, 2010): cytochrome P450 isoforms *CYP1A2* (*1B, rs2470890; *1C, rs2069514; *1F, rs762551), *CYP2A6* (*9, rs28399433), *CYP2B6* (*4, rs2279343; *5, rs3211371; *9, rs3745274; *18, rs28399499; *22, rs34223104; rs4803419), *CYP2C19* (*2, rs4244285; *3, rs4986893; *4, rs28399504; *5, rs56337013; *6, rs72552267; *7, rs72558186; *8, rs41291556; *9, rs17884712; *17, rs12248560; *35, rs12769205), *CYP2C8* (*2, rs11572103; *3, rs10509681 and rs11572080; *4, rs1058930), *CYP2C9* (*2, rs1799853; *3, rs1057910; *5, rs28371686; *8, rs7900194 and rs9332094; *11, rs28371685), *CYP2D6* (*3, rs35742686; *4, rs3892097; *6, rs5030655; *7, rs5030867; *8, rs5030865A; *9, rs5030656; *10, rs1065852; *12, rs5030862; *14, rs5030865T; *15, rs77467110; *17, rs28371706; *19, rs72549353; *29, rs59421388; *41, rs28371725; *56, rs72549347; *59, rs79292917; rs1135840), *CYP3A4* (*2, rs55785340; *3, rs4986910; *6, rs4646438; *18, rs28371759; *22, rs35599367), *CYP3A5* (*3, rs776746; *6, rs10264272; *7, rs41303343), and *CYP4F2* (*3, rs2108622); transporters such as *ABCB1* (C1236T, rs1128503; C3435T, rs1045642; G2677T/A, rs2032582), *ABCC2* (rs2273697), *ABCG2* (rs2231142), *SLC22A1* (*2, rs72552763; *3, rs12208357; rs34059508), *SLC28A3* (rs7853758), and *SLC O 1B1* (*1B, rs2306283; *2, rs56101265; *5, rs4149056; *6, rs55901008; *9, rs59502379; *10, rs56199088; *13, rs56061388; *17/*21, rs4149015; rs11045879); and other drug metabolizing enzymes such as *UGT1A1* (*6, rs4148323; *80, rs887829). A *CYP2D6* copy number variation assay (CNV) was performed in the same thermal cycler with a 96-well thermal block, performed with TaqMan® technology as previously described (Belmonte et al., 2018).

### Haplotyping and Phenotyping

Genotypes were used to infer haplotypes which define phenotypes or diplotypes. The genotyping technique used does not allow knowing with complete certainty whether or not two polymorphisms are located on the same chromosome. This is important in order to correctly define alleles. However, the location of these polymorphisms can be inferred with sufficient confidence from the allele frequency data available. Consequently, allele assignment was conducted according to Clinical Pharmacogenetics Implementation Consortium (CPIC) guidelines for *CYP2C9* and nonsteroidal anti-inflammatory drugs (Caudle et al., 2014), *CYP2C19* and voriconazole (Moriyama et al., 2017), *CYP2D6* and opioids (Crews et al., 2021), *CYP3A5* and tacrolimus (Birdwell et al., 2015), *SLC O 1B1* and simvastatin (Ramsey et al., 2014, 1), and *UGT1A1* and atazanavir (Gammal et al., 2016, 1). The possible phenotypes were ultrarapid (UM), rapid (RM), normal (NM), intermediate (IM) and poor metabolizer (PM) for drug-metabolizing enzymes, and normal (NF) and intermediate function (IF) for transporters. *CYP3A5* phenotype can be denoted either by using the CPIC nomenclature, namely, NM, IM, and PM, or by using the traditional nomenclature of *CYP3A5* “expressors” and “nonexpressors.” In this work, the CPIC nomenclature is used to be consistent with the rest of the genes. NMs are equivalent to expressors (i.e., *1/*1); IMs are equivalent to heterozygotes with one expressor allele (i.e., *1) and one nonexpressor allele (i.e., *3, *6, and *7), and PMs are nonexpressors (i.e., *3/*3 and *3/*6). *CYP2D6* phenotype that resulted ambiguous after CNV (e.g., *1/*4 individuals with three copies that could be interpreted as *1 × 2/*4 or NM and *1/*4 × 2 or IM) was excluded from the analysis. Despite UGT1A1*80 function is unknown, it is in very high linkage disequilibrium with *28, which are decreased function variants. Thus, *1/*1 individuals were considered NMs, *1/*80 subjects were considered IMs, and*80/*80 individuals were considered PMs. *CYP2C8* allele functionality is not defined. Thus, individuals were grouped into diplotypes. For *ABCB1*, following a similar methodology previously published (Zubiaur et al., 2021), individuals were grouped according to their total number of mutations: group 1 was considered any individual with no allelic variants, group 2 consists of those with 1–3 allelic variants, and group 3 consists of those with 4–6 allelic variants. Otherwise, genetic variants were individually analyzed for each gene. The reference SNP number (rs) was named, when available, following the allelic nomenclature following the PharmVar nomenclature [Pharmacogene Variation Consortium (PharmVar), 2018 at www.PharmVar.org (Gaedigk et al., 2018, CPT 103:399; Gaedigk et al., 2019, CPT 105:29)]. A summary table of the correspondence between diplotypes and phenotypes is provided in Supplementary Table S1.

### Safety

During hospitalization, volunteers were asked about treatment tolerability in several occasions. Adverse events (AEs) reported after open questions as well as self-reported AEs were registered in volunteers’ data collection logbook. The causality between drug administration and the occurrence of adverse events (AEs) was evaluated following Karch–Lasagna (Karch and Lasagna, 1977) algorithm for S1 and the algorithm of Spanish Pharmacovigilance System (Aguirre and García, 2016) for S2 and M clinical trial. Only definite, probable, or possible adverse events were considered adverse drug reactions (ADRs) and counted for the present study.

### Statistical Analysis

From 76 initial polymorphisms, 19 final genetic variables were tested (17 genes, but three *CYP1A2* alleles were analyzed independently). Race and clinical trial were added as covariates, and dose/weight correction was applied as control confounding variables. Hardy–Weinberg equilibrium was calculated by χ^2^ test comparing observed and expected allele distributions. Regarding the pharmacokinetics analysis, Vd and Cl were adjusted for bioavailability (i.e., divided by weight) becoming Vd/F and Cl/F, respectively. AUC_∞_, AUC_τ_, C_max_, and C_min_ were adjusted for the dose-weight ratio (DW). Tamsulosin data were obtained both from multiple- and single-dose studies. Since AUC_∞_ after a single dose and AUC_τ_ are equivalent (i.e., they correspond to the total AUC resulting from a drug administration), both variables were merged into a single “AUC” variable. Normality was analyzed by quantile–quantile plots. Homoscedasticity was tested by Levene’s test. For homoscedastic normal variables, differences in means were studied by *t*-test (two categories within a variable) or ANOVA (three or more categories within a variable) with logarithmically transformed pharmacokinetic parameters (e.g., LnAUC), in order to achieve normal distribution. For those variables with three or more groups, a pairwise comparison Bonferroni post hoc analysis was performed. For heteroscedastic variables, differences in means were studied by Welch’s *t*-test (two categories within a variable) or Welch’s ANOVA (three or more categories within a variable). A multivariate analysis was performed by means of linear regression. The significant variants from the univariate analysis and the study design were considered the independent variables for the multivariate analysis of all pharmacokinetic parameters, which were established as dependent variables. Benjamini and Hochberg correction for multiple comparisons was performed, i.e., false discovery rate (FDR) after multivariate analysis (Benjamini and Hochberg, 1995) for 61 tests for tamsulosin and 44 for dutasteride. *p* values lower than 0.05 after FDR correction were considered statistically significant; *p* values lower than 0.05 before FDR correction were considered nominally significant. Concerning treatment safety, the incidence of ADRs depending on phenotypes, genotypes, self-reported race, and clinical trial design was analyzed by χ^2^ test, and the risk of developing those ADRs was calculated by logistic regression. For the ANOVA or t-test, the *p* value is shown for nominally significant relationships (*p*_ANOVA_). For the multivariate analysis, significance (*p* < 0.05) was indicated with the unstandardized *β*-coefficient, R^2^ value, *p* of multivariate analysis (p_MV_), and *p* after FDR (*p*_FDR_). All calculations were computed in R version 4.0.3 software (R Core Team, 2020).

### Ethics

The protocol and informed consent for the three clinical trials were approved by the Independent Ethics Committee (IECCR, CEIm) of Hospital Universitario de la Princesa and the Spanish Drug Agency (AEMPS). S1 (EUDRA-CT number: 2017-001592-23), S2 (EUDRA-CT number: 2017-003227-29), and M (EUDRA-CT number: 2017-003244-21) clinical trials were performed according to Spanish regulation, ICH guidelines for Good Clinical Practices, and Revised Declaration of Helsinki (World Medical Association, 1991).

## Results

### Demographic Results

The study population comprised 79 male healthy volunteers, defined by mean ± standard deviation, with a median age of 24 ± 6.7 years old, mean height of 1.76 ± 0.07 m, mean weight of 76.87 ± 8.72 kg, and body max index (BMI) of 24.86 ± 2.26 m/kg^2^. The population was composed of 52 (74%) Caucasians and 18 (26%) Latin individuals. No significant differences in demographics were found between these two groups.

All polymorphisms analyzed were in Hardy–Weinberg equilibrium, except for *CYP1A2* *1C (rs2069514), *CYP2A6* *9 (rs28399433), *CYP2B6* *4 (rs2279343), *ABCB1* rs2032582, *CYP2C8* *8 (rs1058930), and *CYP3A4* *22 (rs35599367).

### Dutasteride

All the analyzed variables presented normal distributions after logarithmic adjustment. All variables presented homoscedastic distribution except t_max_ for *CYP2A6* *9 and *SLC22A1* *2, t_1/2_ for *CYP1A2* *1B and *CYP2C9* phenotype, and Vd/F for *CYP2C9* phenotype.

Fed conditions presented higher t_max_ (*p*_ANOVA_ = 0.002) and higher Vd/F than fasting conditions (*p*_ANOVA_ = 0.006) after univariate analysis. Moreover, CYP3A4*22 allele carriers showed lower Vd/F than *1/*1 individuals (*p*_ANOVA_ = 0.023). Additionally, *SLC28A3* rs7853758 A/G and A/A subjects presented lower AUC (*p*_ANOVA_ = 0.012) and higher Cl/F (*p*_ANOVA_ = 0.043) than G/G. *SLC28A3* rs7853758 (*β* = −0.51, R^2^ = 0.15, *p*_MV_ = 0.011, and *p*_FDR_ = 0.065) maintained significance in multivariate analysis for AUC. Food (*β* = 0.3, R^2^ = 0.39, *p*_MV_ = 0.016, and *p*_FDR_ = 0.087), *CYP3A4* genotype (*β* = −0.7, *p*_MV_ = 0.024, and *p*_FDR_ = 0.11), and *SLC28A3* rs7853758 (*β* = 0.26, R^2^ = 0.39, *p*_MV_ = 0.039, and *p*_FDR_ = 0.16) remained significant after multivariate analysis for Vd/F. Nonetheless, all of these variables lost significance after FDR correction (Table 1). Thus, no statistically significant effect was found for dutasteride.

**TABLE 1:** Significant relationships between dutasteride pharmacokinetics and clinical trial design, volunteers self-reported race, and genotype.

			AUC (ng·h/mL)	C_max_ (ng/ml)	t_max_ (h)	t_1/2_(h)	Vd/F (L/kg)	Cl/F (ml/h·kg)
SRR		Caucasian (*n* = 38)	44.21 (49.22%)	2.49 (37.21%)	2.74 (57.1%)	61.95 (45.03%)	9.39 (66.19%)	138.42 (103.53%)
		Latin (*n* = 14)	42.87 (45.69%)	2.56 (27.41%)	3.68 (48.16%)	49.36 (41.09%)	7.58 (34.56%)	144.97 (93.54%)
CT		Fed (S1) (*n* = 25)	49.53 (48.12%)	2.76 (30.37%)	3.68 (44.53%)* ^**1**^	56.25 (47.96%)	7.32 (50.29%)* ^**1**^ **†**	142.22 (120.99%)
		Fasting (S2) (*n* = 27)	38.59 (43.54%)	2.28 (36.86%)	2.35 (60.65%)	60.7 (43.4%)	10.37 (62.86%)	138.3 (76.17%)
*CYP3A4*		*1/*1 (*n* = 50)	42.17 (43.51%)	2.49 (32.62%)	2.98 (55.49%)	58.56 (46.14%)	9.09 (60.9%)	143.79 (98.44%)
		*1/*22 + *22/*22 (*n* = 2)	85.76 (56.8%)	3.11 (74.06%)	3.25 (76.15%)	58.54 (1.52%)	4.23 (47.72%)* ^**1**^ **†**	49.91 (46.37%)
*CYP3A5*		NM + IM (*n* = 9)	38.17 (52.17%)	2.17 (33.36%)	3.89 (49.7%)* ^**1**^	45.04 (27.19%)	8.81 (42.07%)	168.31 (92.21%)
		PM (*n* = 43)	45.04 (47.23%)	2.58 (34.25%)	2.8 (55.49%)	61.39 (45.37%)	8.92 (65.65%)	134.3 (102.64%)
*SLC28A3*	rs7853758	A/A (*n* = 4)	37.33 (67.95%)	2.65 (35.92%)	2 (28.87%)	55.52 (48.76%)	9.65 (42.55%)	207.06 (115.38%)
		A/G (*n* = 14)	35.51 (57.83%)	2.26 (39.61%)	2.91 (51.24%)	50.08 (46.7%)	11.03 (77.17%)	200.91 (94.4%)
		G/G (*n* = 31)	49.27 (39.89%)* ^**2**^ **†**	2.63 (31.92%)	3.16 (57.37%)	63.6 (43.32%)	**7.64 (34.9%)†**	98.26 (63.7%)* ^**2**^

Data is presented as mean (coefficient of variation). SRR: Self-reported race, CT: Clinical Trial, S1: single-dose feeding conditions trial, S2: single-dose fasting conditions trial, NM: Normal metabolizer, IM: Intermediate metabolizer, PM: Poor metabolizer. *^1^: *p* < 0.05 after ANOVA, *^2^: *p* < 0.05 after ANOVA and Bonferroni post-hoc vs A/G, †: nominal *p* < 0.05 after multivariate analysis. No variable remained statistically significant after FDR correction. Bold data represents significant results.

### Tamsulosin

All the analyzed variables presented normal distributions after logarithmic adjustment. All variables presented homoscedastic distribution, except: AUC and Cl/F for *CYP2A6* *9; Vd/F for CYP1A2*1F and *CYP2A6* *9; t_1/2_ for CYP1A2*1F, SLC22A1*3, and *CYP2C9* phenotype; and C_max_ for clinical trial design and *CYP2C9* phenotype.

Fasting conditions presented higher AUC (*p*_ANOVA_ = 0.011) than fasting and multiple dose. Fasting conditions and multidose administration exhibited lower t_max_ (*β* = −0.21, R^2^ = 0.16, *p*_MV_ = 0.001, and *p*_FDR_ = 0.008) and higher C_max_ (*β* = 0.22, R^2^ = 0.25, *p*_MV_ = 0.014, and *p*_FDR_ = 0.063) than fed conditions. Moreover, multivariate analysis also revealed that single-dose administration showed lower Vd/F (*β* = −0.22, R^2^ = 0.31, *p*_MV_ = 0.002, and *p*_FDR_ = 0.008) and lower t_1/2_ (*β* = −0.20, R^2^ = 0.30, *p*_MV_ = 0.001, and *p*_FDR_ = 0.008) than single-dose trials. *ABCG2* rs2231142 C allele carriers presented higher Vd/F (*p*_ANOVA_ = 0.014) than G/G individuals. Univariate and multivariate analysis also revealed that *CYP2D6* UMs and NMs presented lower AUC than PMs and IMs (*β* = −0.34, R^2^ = 0.36, *p*_MV_<0.001, and *p*_FDR_ = 0.004). Additionally, UMs and NMs had lower t_1/2_ (*β* = −0.20, R^2^ = 0.30, *p*_MV_ = 0.002, and *p*_FDR_ = 0.008), higher Vd/F (*β* = 0.14, R^2^ = 0.31, *p*_MV_ = 0.046, and *p*_FDR_ = 0.19), and lower Cl/F (*β* = 0.33, *p*_MV_ = 0.009, and *p*_FDR_ = 0.006) than PMs and IMs. *CYP3A5* NMs and IMs presented higher Vd/F (*p*_ANOVA_ = 0.019) and Cl/F (*p* = 0.027) than PM. Finally, *SLC22A1* *1/*3 individuals presented higher AUC (*p*_ANOVA_ = 0.020), higher C_max_ (*p*_ANOVA_ = 0.017), higher C_min_ (*p*_ANOVA_ = 0.038), and lower Cl/F (*p*_ANOVA_ = 0.026) than *1/*1 volunteers (Table 2).

**TABLE 2:** Significant relationships between tamsulosin pharmacokinetics and clinical trial design, volunteers self-reported race and genotype.

				AUC (ng·h/mL)	C_max_ (ng/ml)	C_min_ (ng/ml)
SRR			Caucasian (*n* = 56)	176.13 (47.37%)	12.28 (36.83%)	3.26 (65.53%)
			Latin (*n* = 23)	160.16 (44.27%)	12.84 (37.39%)	2.43 (51.48%)
CT			Fed (S1) (*n* = 25)	148.51 (49.05%)	10.35 (36.44%)* ^**2**^	—
			Fasting (S2) (*n* = 27)	202.57 (47.75%)* ^**1**^	13.85 (41.83%)	—
			Multiple dose (M) (*n* = 27)	161.66 (35.39%)	12.98 (24.04%)	2.98 (63.78%)
*ABCG2*	rs2231142		G/G (*n* = 65)	177.70 (47.41%)	12.67 (36.58%)	3.17 (65.42%)
			G/T (*n* = 14)	142.62 (33.15%)	11.38 (38.01%)	2.33 (42.27%)
*CYP2D6*			UM (*n* = 11)	131.02 (28.5%)	10.37 (27.41%)	1.81 (51.59%)
			NM (*n* = 37)	145.63 (41.7%)	11.53 (34.92%)	2.54 (49.92%)
			IM (*n* = 23)	221.00 (43.3%)* ^**5**^ **†** ^**3**^ **‡**	14.87 (36.24%)	3.38 (35.58%)
			PM (*n* = 6)	**223.07 (36.11%)†** ^**3**^ **‡**	12.66 (36.2%)	5.11 (90.43%)
*CYP3A5*			NM + IM (*n* = 16)	139.47 (49.29%)	10.59 (33.14%)	2.23 (47.41%)
			PM (*n* = 63)	179.61 (45.06%)	12.91 (36.59%)	3.24 (64.06%)
*SLC22A1*	rs12208357		*1/*1 (*n* = 73)	165.11 (45.47%)	12.07 (36.95%)	2.77 (69.1%)
			*1/*3 (*n* = 6)	248.97 (41.24%)* ^**4**^	17.01 (22.15%)* ^**4**^	4.68 (4.14%)* ^**4**^

			t_max_ (h)	t_1/2_(h)	Vd/F (ml/kg)	Cl/F (ml/h·kg)
SRR	Caucasian (*n* = 56)		6.41 (25.16%)	11.94 (32.55%)	556.69 (29.83%)	109.93 (43.07%)
	Latin (*n* = 23)		6.52 (25.58%)	11.7 (23.15%)	617.16 (38.7%)	115.7 (42.44%)
CT	Fed (S1) (*n* = 25)		7.36 (27.91%)* ^**2**^ **†** ^**1**^ **‡**	10.04 (22.95%)	557.64 (29.69%)	127.18 (42.17%)
	Fasting (S2) (*n* = 27)		5.83 (21.13%)	11.86 (32.09%)	498.61 (31.98%)* ^**1**^ **‡**	97.44 (44.79%)* ^**1**^
	Multiple dose (M) (*n* = 27)		6.2 (17.64%)	13.58 (26.10%)* ^**3**^ **†** ^**2**^	665.4 (31.28%)	111.36 (38.13%)
*ABCG2*	rs2231142	G/G (*n* = 65)	6.21 (29.56%)	11.87 (29.34%)	548.25 (30.13%)	107.63 (40.83%)
		G/T (*n* = 14)	6.49 (24.33%)	11.89 (34.41%)	695.21 (36.49%)* ^**4**^	130.09 (46.48%)
*CYP2D6*	UM (*n* = 11)		6.14 (24.2%)	10.42 (23.81%)	667.2 (34.99%)	137.35 (39.51%)
	NM (*n* = 37)		6.55 (28.7%)	10.85 (24.58%)	595.42 (31.71%)	123.68 (39.44%)
	IM (*n* = 23)		6.35 (20.49%)	12.89 (23.56%)* ^**5**^ **†** ^**3**^ **‡**	491.02 (30.97%)* ^**5**^ **†** ^**3**^	86.76 (38.68%)* ^**5**^ **†** ^**3**^ **‡**
	PM (*n* = 6)		7 (18.07%)	**16.98 (36.68%)†** ^**3**^ **‡**	**577.06 (25.43%)†** ^**3**^	**83.69 (39.13%)†** ^**3**^ **‡**
*CYP3A5*	NM + IM (*n* = 16)		6.75 (27.18%)	11.4 (23.38%)	686.97 (34.88%)*	137.7 (41.26%)*
	PM (*n* = 63)		6.37 (24.61%)	11.99 (31.46%)	545.68 (30.48%)	104.98 (41%)
*SLC22A1*	rs12208357	*1/*1 (*n* = 73)	6.47 (25.85%)	11.73 (30.76%)	583.65 (32.68%)	114.3 (41.3%)
		*1/*3 (*n* = 6)	6.17 (12.21%)	13.57 (20.12%)* ^**4**^	460.44 (34.76%)* ^**4**^ **†** ^**4**^	78.81 (55.1%)* ^**4**^ **†** ^**4**^

Data are presented as mean (coefficient of variation). SRR: Self-reported race, CT: Clinical Trial, S1: single-dose feeding conditions trial, S2: single-dose fasting conditions trial, M: multiple-dose feeding conditions trial, UM: Ultrarrapid metabolizer, NM: Normal metabolizer, IM: Intermediate metabolizer, PM: Poor metabolizer.*^1^: *p* < 0.05 after ANOVA and Bonferroni post-hoc analysis vs S2 vs M. *^2^: *p* < 0.05 after ANOVA and Bonferroni post-hoc analysis vs S2 vs M. *^3^: *p* < 0.05 after ANOVA and Bonferroni post-hoc analysis vs S1 vs S2. *^4^: *p* < 0.05 after ANOVA. *^5^: *p* < 0.05 after ANOVA and Bonferroni post-hoc analysis vs UM and NM. †^1^: nominal *p* < 0.05 after multivariate analysis vs S2 and M. †^2^: nominal *p* < 0.05 after multivariate analysis vs S1 and S2. †^3^: nominal *p* < 0.05 after multivariate analysis vs UM and NM. †^2^: nominal *p* < 0.05 after multivariate analysis vs S1 and S2. †^3^: nominal *p* < 0.05 after multivariate analysis vs UM and NM. †^2^: nominal *p* < 0.05 after multivariate analysis vs S1 and S2. †^4^: nominal *p* < 0.05 after multivariate analysis ‡: *p* < 0.05 after FDR. C_min_ was only calculated in M clinical trial. Bold data represents significant results.

After FDR, the *CYP2D6* phenotype remained statistically significant for tamsulosin AUC, Cl/F, t_1/2_, and t_max_, and clinical trial design remained the statistically significant variables for tamsulosin Vd/F, t_1/2_, and t_max_.

### Safety

No serious ADR was reported. The ADRs reported comprised dizziness, testicular pain, epididymo-orchitis, headache, ejaculation disorder, hypotension symptomatic, retrograde ejaculation, libido decreased, and abnormal urine odor. Eight volunteers presented at least one ADR. The most frequent ADRs were headache (*n* = 3) and retrograde ejaculation (*n* = 3), followed by libido decrease (*n* = 2) and ejaculation disorder (*n* = 2). The remaining ADRs were only observed in one volunteer. Participants in the multiple-dose clinical trial were related to higher incidence of ADR than participants in single-dose (7 ADR vs. 1 ADR, respectively; *p* < 0.05). No relationship between polymorphisms or race with ADR occurrence was found.

Nonsignificant results are provided in Supplementary Table S2.

## Discussion

Dutasteride and tamsulosin are widely used drugs effective for the treatment of BPH. However, drug underexposure can lead to a lack of effectiveness, and overexposure, to the occurrence of ADRs; as mentioned earlier, both circumstances may lead to drug discontinuation. In order to achieve safe and effective responses to pharmacological treatments, pharmacogenetic-based dose adjustments are proposed for different drugs by institutions such as CPIC (Amstutz et al., 2018; Crews et al., 2021) and DPGW (Dutch Pharmacogenetics Working Group Pharmacogenetic Recommendations and 2019) or regulatory agencies such as FDA or EMA. In particular, FDA and EMA drug labels for tamsulosin 0.4 mg and combined formulations (e.g., Duodart®) warrant precaution for *CYP2D6* PMs using concomitant *CYP3A4* inhibitors (FDA, 2010b; Agencia Española del Medicamento y Productos Sanitarios (AEMPS), 2015). Subjects with this phenotype may be overexposed to tamsulosin, and ADRs may occur. Nonetheless, no additional pharmacogenetic guideline or dose adjustment recommendation is available for tamsulosin. Neither is there any prescribing information available for dutasteride. The latter is consistent with the scarcity of well-designed observational pharmacogenetic studies for both drugs, especially for dutasteride. Thus, our intention in this study is to further elucidate the effects of pharmacogenetics on these two drugs.

The observed dutasteride pharmacokinetic parameters were in general congruent with the literature, e.g., AUC of 39.6 ± 23.1 ng·h/ml and C_max_ of 2.14 ± 0.77 ng/ml, compared with 43.03 ± 20.73 ng·h/ml and of 2.46 ± 0.89 ng/ml, respectively (Agencia Española del Medicamento y Productos Sanitarios (AEMPS), 2015). No significant difference was found between the two groups of race and any pharmacokinetic parameter.

Feeding is important for absorption velocity of orally administered drugs. Meals (especially high-fat meals) delay gastric emptying, augmenting the transit time to the small intestine and, subsequently, delaying the absorption into the systemic circulation (McLachlan and Ramzan, 2006). As expected, fed individuals presented higher dutasteride t_max_ than fasting volunteers and lower Vd/F. Despite not being statistically significant, a 34% higher AUC and 22% higher C_max_ were observed in fed individuals compared to fasting volunteers, which is consistent with the nominally significant differences observed in the Vd/F. Nonetheless, these results did not remain significant after multiple testing corrections, which is congruent with previous bioequivalence clinical trials that reported no differences in dutasteride pharmacokinetics (Kurczewski et al., 2017).

Consistent with the well-known pharmacokinetic profile of dutasteride, pharmacokinetic variability was significantly related to *CYP3A4* and *CYP3A5* polymorphism (Agencia Española del Medicamento y Productos Sanitarios (AEMPS), 2015). *CYP3A4* *22 allele carriers showed more than double AUC than *1/*1 carriers. However, this difference was not significant, likely due to the reduced number of volunteers carrying the *22 allele (*n* = 2). Similar to the explanation for the feeding conditions, these volunteers consistently presented significantly lower Vd/F. Moreover, *CYP3A5* NMs and IMs showed a higher t_max_ compared to PMs. This might reflect a reduced rate of elimination by PMs. As the elimination rate decreases, it requires less time for the drug to accumulate and to reach peak concentration. Consistently, we observed a 30% greater t_1/2_ in PMs compared to NM + IMs (however, this difference was not statistically significant). However, as these results did not remain significant after multiple testing corrections, they might be spurious. Lastly, *SLC28A3* rs7853758 A/G and A/A subjects presented lower AUC, lower t_1/2_, and higher Cl/F than G/G. Nonetheless, none of these associations remained significant after FDR correction. Further studies are warranted to confirm whether *CYP3A* or *SLC28A3* polymorphism affects dutasteride pharmacokinetics.

The observed tamsulosin pharmacokinetic parameters under single-dose after fed conditions were similarly consistent with the literature, for example, AUC of 187.2 ± 95.7 ng·h/ml and C_max_ of 11.3 ± 4.44 ng/ml compared to 147.4 ± 72.8 ng·h/ml and 10.35 ± 3.77 ng/ml, respectively (Agencia Española del Medicamento y Productos Sanitarios (AEMPS), 2015) (JALYN). No significant difference was found between the two groups of race and any pharmacokinetic parameter.

As mentioned before, food alters drug absorption and, therefore, pharmacokinetic parameters linked to it (e.g., t_max_ or C_max_). This is likely caused by the different solubility of a drug based on the stomach pH and the transit time to the small intestine. Previous works state that fasting conditions are related to faster and greater tamsulosin absorption (FDA, 1997). Consistently, in this work, fasting volunteers exhibited nominally significant higher AUC (29%) and lower t_max_ (15%) compared to fed volunteers. Congruent with literature, a 16% higher C_max_ was also observed in fasting volunteers; nonetheless, the association was not significant (Agencia Española del Medicamento y Productos Sanitarios (AEMPS), 2015). As expected, we found a statistically significant higher t_max_ when the drug was administered after a high-fat breakfast, compared to fasting conditions. Additionally, as expected, fed administration had lower C_max_ than multiple-dose administration. Finally, the nonexistent difference between the AUC of fed and multiple dose is consistent because, under the same conditions, the AUC_∞_ is equivalent to the AUC_τ_. Multiple-dose t_1/2_ was found significantly higher than fed t_1/2_. Theoretically, for drugs with linear pharmacokinetics like tamsulosin, t_1/2_ should remain constant regardless of the dose or administration regimen. However, we observed a greater t_1/2_ in multiple dose compared to fed conditions. This difference is likely explained by the limitations of noncompartmental analysis and the possibility of a type-1 error. Nevertheless, both t_1/2_ values coincided with the range provided in the literature (10–13 h) (Agencia Española del Medicamento y Productos Sanitarios (AEMPS), 2015).

Tamsulosin is 90% metabolized by *CYP3A4* and *CYP2D6*, but also by other cytochrome P450 isoforms to a lesser extent (Agencia Española del Medicamento y Productos Sanitarios (AEMPS), 2015). Previous studies reported a relationship between tamsulosin bioavailability and *CYP2D6* phenotype (Choi et al., 2012; Byeon et al., 2018; Kim et al., 2018). Our results confirm that tamsulosin pharmacokinetics is significantly altered by the *CYP2D6* phenotype: PMs and IMs exhibited a significantly higher bioavailability than NMs and UMs. Although we did not observe differences in ADR incidence due to the limitations in our study design and we had no effectiveness data, our results indicate that UMs will likely be underexposed and PMs overexposed leading to ineffectiveness and worse tolerability, respectively. Considering that ADR occurrence was significantly related to the multiple dose clinical trial, we can assume that an enhanced exposure to the drug relates to a higher incidence of ADRs. We suggest that a dose reduction for PMs or an increase for UMs could be beneficial. However, further studies are required to indicate the extent of such dose modifications. The only formulation strength available for tamsulosin (in combination) is 0.4 mg, both in Europe and United States (EMA and FDA, respectively) (FDA, 2010b; Agencia Española del Medicamento y Productos Sanitarios (AEMPS), 2015). There is, therefore, a need for the marketing of formulations that facilitate the individualization of pharmacotherapy (e.g., dutasteride-tamsulosin 0.5/0.3 mg and 0.5/0.5 mg strengths). Nevertheless, further studies are warranted to confirm the clinical relevance of our conclusions. Whether patients may benefit or not from dose adjustments based on *CYP2D6* phenotype should be demonstrated prior to routine implementation. Entities such as CPIC, SEFF, or DPWG may eventually publish clinical guidelines supporting or rejecting the need for a pharmacogenetic-guided prescription.

*CYP3A5* PMs showed lower Cl/F than NMs and IMs. Considering that tamsulosin is a *CYP3A4* substrate, it would be expected that *CYP3A5* metabolized it. This association suggests that tamsulosin is a *CYP3A5* substrate and that its phenotype contributes to its pharmacokinetic variability. However, previous research studies (FDA, 2010b; Agencia Española del Medicamento y Productos Sanitarios (AEMPS), 2015) reported no relationship between *CYP3A5* genotype and tamsulosin pharmacokinetic variability (Kim et al., 2018). Moreover, the association lost significance after applying FDR correction. To the best of our knowledge, this is the first work to suggest a similar association. However, further studies are required to replicate our observation.

*ABCG2* encodes for the Breast Cancer Resistant Protein (BCRP). It is an ATP-binding cassette transporter and plays a major role in multidrug resistance, specially involved in the response to mitoxantrone and anthracycline (Bethesda, 2004). The impact of rs2231142 is controversial. T/T individuals were associated with decreased clearance of sulfasalazine in healthy individuals as compared to genotypes GG + GT (Gotanda et al., 2015). In this study, conversely, G/T individuals presented a significantly higher Vd/F than G/G individuals and approximately 18% lower AUC (not significant) and 23% higher Cl/F (not significant). This suggests, on the contrary, that tamsulosin is a BCRP substrate and that rs2231142 is related to lower exposure. As these results did not remain significant after multiple testing corrections, further studies should investigate the impact of this polymorphism and whether tamsulosin is a BCRP substrate.

*SLC22A1* encodes for the organic cationic transporter 1 (OCT1), one of the three similar polyspecific cationic transporters mediating the uptake of many organic cations from the blood. It has substrate selectivity for a variety of endogenous ligands (dopamine, serotonin, and choline) as well as cationic drugs, such as metformin, cimetidine, imatinib, oxaliplatin, and tramadol and agmatine. OCT1 carries drugs into the liver and kidneys, where the compound is metabolized and excreted (Whirl-Carrillo et al., 2012). *SLC22A1* *1/*3 individuals presented significantly higher AUC, C_max_, C_min_, t_1/2_, and lower Vd/F and Cl/F than *1/*1. This suggests that tamsulosin might be an OCT1 substrate. The potential reduced function of the transporter could reduce drug’s hepatic uptake and, consequently, the elimination of tamsulosin, thus incrementing its bioavailability. Considering that this association did not remain significant after FDR correction, these findings could be considered spurious. Nevertheless, further studies would be necessary to confirm if tamsulosin pharmacokinetics is impacted by *SLC22A1* polymorphism.

Nonetheless, this study presents several limitations. First, the sample size is small. To address this issue, three different clinical trials were analyzed. This leads to the second limitation: merging of three different study designs complicates the statistical analysis. The study design was analyzed as a covariate, but despite this, the statistical power is more limited than in a unique study design. Furthermore, the incidence of *CYP2D6* UMs is significantly higher than expected from literature (14 vs. 7%, respectively). We are confident with the robustness of our genotyping, but we must note this limitation. Thus, further studies are required to confirm the results here obtained.

## Conclusion

*CYP2D6* phenotype severely affected tamsulosin pharmacokinetics. PMs and IMs presented twice higher exposure to tamsulosin than UMs and NMs. The results were consistent with the literature and the guidelines of regulatory institutions, such as FDA and EMA, which do not include specific dose adjustment recommendations. Here, we suggest that a dose adjustment could improve tamsulosin effectiveness and safety. Further studies are warranted to confirm whether this adjustment would be beneficial for the patient. Alternatively, dutasteride pharmacokinetics was not altered based on genotypes or drug dose regimen. To the best of our knowledge, this is the largest study analyzing tamsulosin pharmacogenetics (*n* = 79) and the first study of this type for dutasteride. Additionally, new potential associations were proposed regarding *ABCG2, CYP3A4, CYP3A5*, and *SLC22A1*. However, the main limitation of this study is the limited sample size. Consequently, further prospective studies should be addressed to confirm such associations.

## Data Availability

The raw data supporting the conclusion of this article will be made available by the authors, without undue reservation. The data is the property of the promoter and will be made available upon reasonable request.
