# Supplementary material for: Effects of Cytochrome P450 and Transporter Polymorphisms on the Bioavailability and Safety of Dutasteride and Tamsulosin
Source: Front Pharmacol. 2021 Oct 7;12:718281. doi: 10.3389/fphar.2021.718281 (PMC8529037; doi:10.3389/fphar.2021.718281)
Supplement: Supplementary file 1 [file DataSheet1.docx]

Supplementary Material

# Supplementary Figures and Tables

##
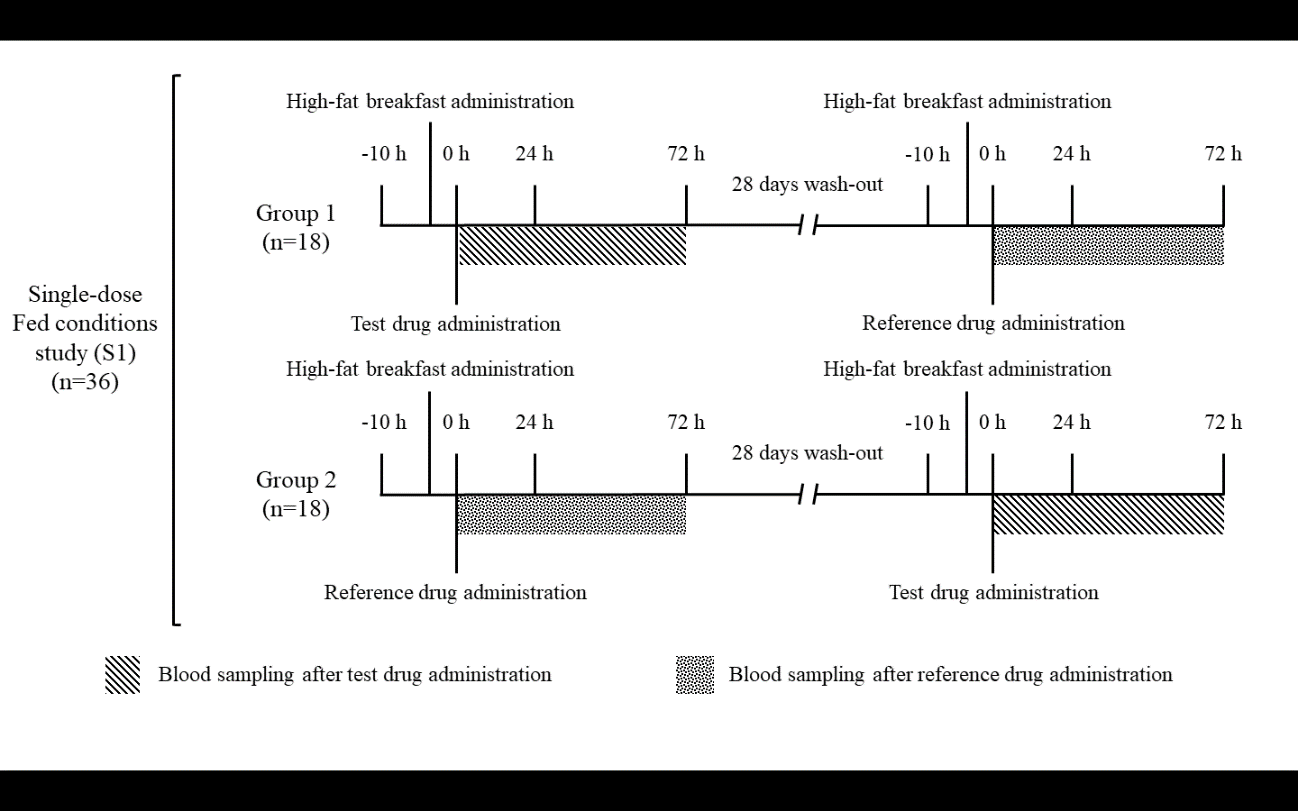
Supplementary Figures

**Figure 1.** Diagram of the study design of the single-dose under fed conditions bioequivalence clinical trial (S1). The diagonal stripe pattern corresponds to sampling time after the volunteers received the test formulation. The dotted pattern corresponds to sampling time after the volunteers received the reference formulation.


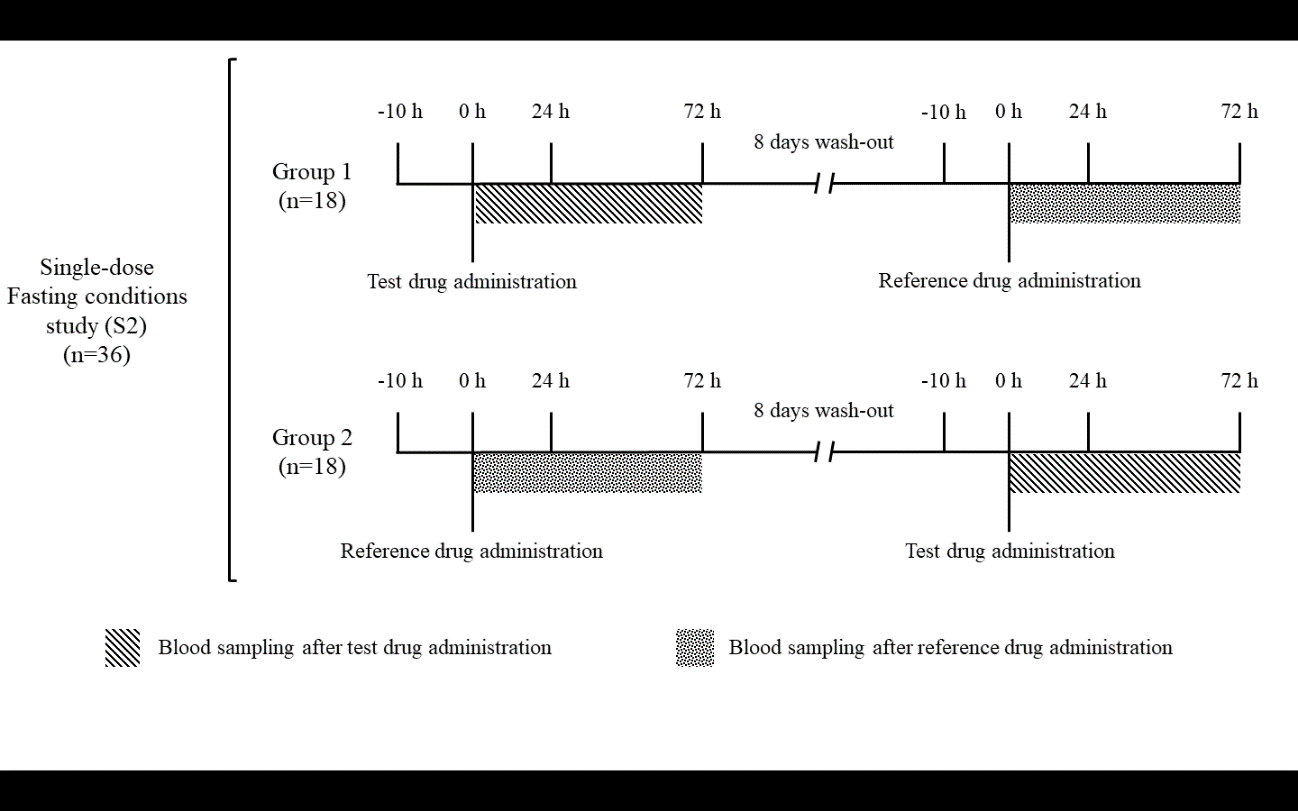

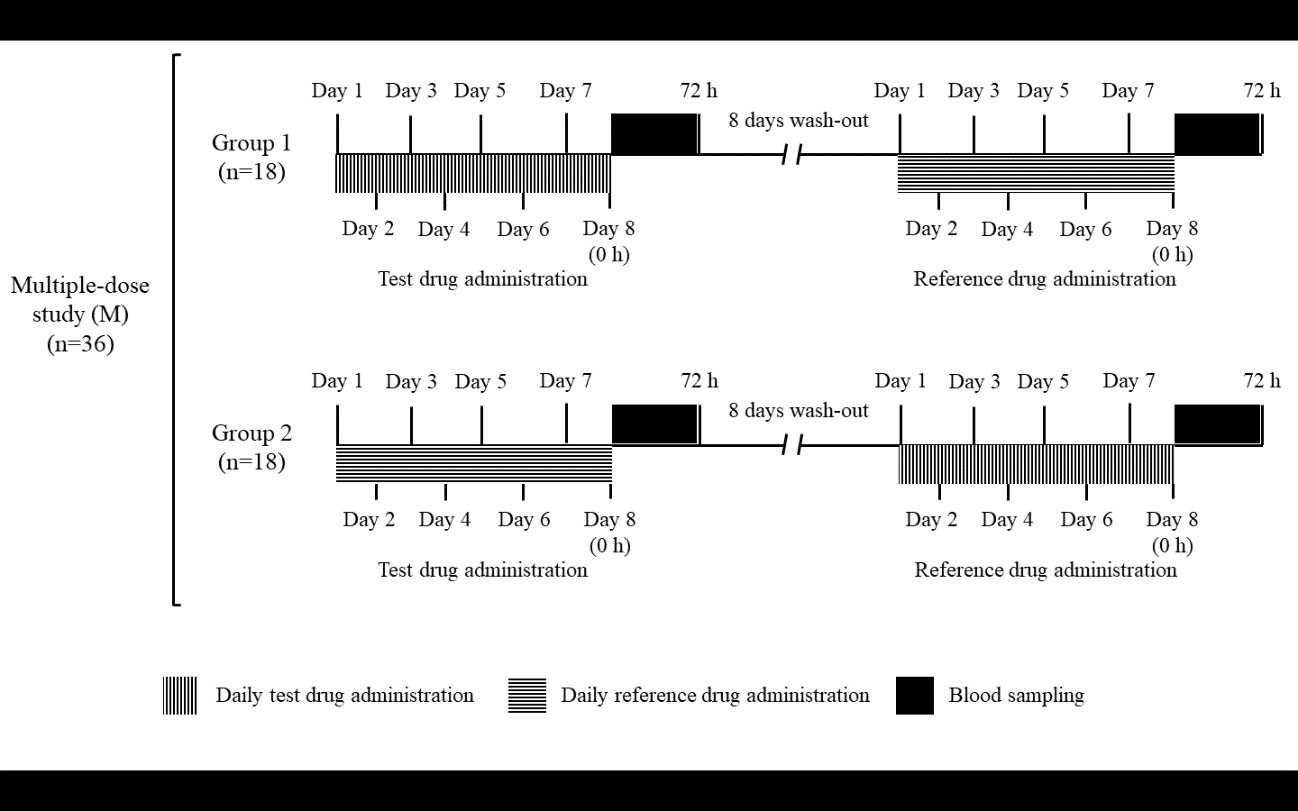
**Figure 2.** Diagram of the study design of the single dose under fasting conditions bioequivalence clinical trial (S2). The diagonal stripe pattern corresponds to sampling time after the volunteers received the test formulation. The dotted pattern corresponds to sampling time after the volunteers received the reference formulation

**Figure 3**. Diagram of the study design of the multiple dose clinical trial (M). The vertical stripe pattern corresponds to the period when volunteers received the test formulation. The horizontal stripe pattern corresponds to the period when volunteers received the reference formulation. The black pattern corresponds to time of blood sampling.

## Supplementary Tables.

**Suplementary Table 1:** Correspondence of phenotypes and haplotypes found in this study. UM: ultrarapid metabolizer; RM: rapid metabolizer; NM: normal metabolizer; IM: intermediate metabolizer; PM: poor metabolizer; NF: normal function; DF: decreased function.

|  | **Diplotype** | **Phenotype** |  |  | **Diplotype** | **Phenotype** |
| --- | --- | --- | --- | --- | --- | --- |
| **CYP2B6** | *1/*1 | NM |  | **CYP3A5** | *1/*1 | NM |
|  | *1/*4 | RM |  |  | *1/*3 | IM |
|  | *1/*5 | NM |  |  | *3/*3 | PM |
|  | *1/*6 | IM |  |  | *3/*6 | PM |
| **CYP2C19** | *1/*1 | NM |  | **SLCO1B1** | *1/*1 | NF |
|  | *1/*17 | RM |  |  | *1/*15 | DF |
|  | *1/*2 | IM |  |  | *1/*17 | DF |
|  | *2/*17 | IM |  |  | *1//*1B | NF |
| **CYP2C9** | *1/*1 | NM |  |  | *1B/*1B | NF |
|  | *1/*2 | IM |  | **UGT1A1** | *1/*1 | NM |
|  | *1/*3 | IM |  |  | *1/*6 | IM |
|  | *2/*2 | IM |  |  | *1/*80 | IM |
| **CYP2D6** | *1/*1 | NM |  |  | *80/*80 | PM |
|  | *1/*1x2 | UM |  |  |  |  |
|  | *1/*4 | IM |  |  |  |  |
|  | *1/*5 | IM |  |  |  |  |
|  | *4/*15 | PM |  |  |  |  |
|  | *4/*4 | PM |  |  |  |  |
|  | *4/*5 | PM |  |  |  |  |

**Supplementary Table 2**. Data are presented as mean (coefficient of variation). UM: ultrarapid metabolizer, RM: rapid metabolizer, NM: normal metabolizer, IM: intermediate metabolizer, PM: poor metabolizer, NF: normal function; DF: decreased function.

(Attached as a separate document)
